# Exploring the Role of Secondary Metabolites in Post-Fire Ecological Memory

**DOI:** 10.3390/plants15203235

**Published:** 2026-10-21

**Authors:** Zorica Popović, Vera Vidaković

**Affiliations:** Department of Ecology, Institute for Biological Research “Siniša Stanković”—National Institute of the Republic of Serbia, University of Belgrade, Bulevar despota Stefana 142, 11108 Belgrade, Serbia; vera.vidakovic@ibiss.bg.ac.rs

**Keywords:** post-fire, secondary metabolites, phenolics, terpenoids, karrikins, chemical memory

## Abstract

Fire is a significant cause of ecosystem disturbance with many direct and indirect effects on the structure and functioning of plant communities. The effects of fire disturbance and post-fire acclimation have been well-documented through changes in plant community composition and structure and the triggering of multiple adaptive responses. However, the role of plant secondary metabolites (SM) in the post-fire acclimation process is underestimated. These compounds have mostly been recognized as determinants of plant flammability, while their post-fire dynamics and ecological consequences remain largely unknown. This literature review synthesizes evidence from 43 studies on changes in plant SM following wildfires and prescribed burns and their potential ecological roles. Literature searches were conducted in Web of Science and Scopus, and a bipartite co-occurrence network analysis was performed to examine associations among vegetation types, post-fire timelines, and secondary metabolite classes, including the direction and frequency of reported responses. Phenolic compounds and volatile organic compounds (VOCs) were the main SM groups identified, with substantial variation in the direction and magnitude of responses depending on species and time since fire. Karrikins and their potential role in post-fire environments were considered separately as fire-derived signaling compounds. Key knowledge gaps include uneven representation of ecological zones, limited compound-specific analyses, insufficient integration of fire-induced metabolite responses with subsequent ecological effects, and limited translation of biochemical findings into ecosystem management and restoration.

## 1. Introduction

Forest fires represent a key ecological disturbance with profound effects on plant communities and ecosystem functioning across temporal and spatial scales [1,2]. The recovery of a post-fire ecosystem is complex and multilayered, involving interactions among fire severity, community and species traits, environmental conditions, and legacy effects [3,4]. These processes synergistically contribute to ecological memory, defined as the capacity of past states or disturbances to influence present and future ecosystem responses [5,6]. Resilience and recovery trajectories after fire disturbances are shaped by ecological memory, which operates at several organizational levels, from soil characteristics and seed banks to species adaptations and community assembly [5,7].

Substantial biomass loss, along with significant changes in species composition, vegetation structure, and trophic interactions within the ecosystem, typically represents the initial evidence of a fire [8,9]. From a biochemical perspective, studies on the effects of fire have mainly focused on structural cell wall components such as cellulose, hemicellulose, and lignin, which are major constituents of plant biomass [10]. These compounds play a direct role in combustion processes and influence the dynamics of soil organic matter and carbon cycling following a fire.

Plant secondary metabolites are chemical compounds with multiple functions that help balance plant metabolism and ensure optimal responses to environmental challenges [11]. This chemically diverse group is mainly associated with stress signaling and biotic interactions, such as defense against pathogens and herbivores and allelopathy [12,13]. Studies indicate that these compounds may play a major role in how plants respond to environmental stressors by modulating species interactions, regeneration capacity, and post-disturbance succession [14,15]. Certain classes of secondary metabolites have been shown to significantly affect plant flammability, ignition potential, and fire spread [16,17,18]. Research shows that highly volatile terpenoids contribute to rapid ignition in conifers, while phenolics and tannins influence litter combustibility and thermal stability (e.g., [17,19,20,21]).

In contrast to extensive research on their functional role in fire initiation and progression, the post-fire dynamics of plant secondary metabolites are poorly characterized. Research indicates that certain types of secondary metabolites change in quantity after fire events [22,23,24,25]. However, fewer studies have examined whether qualitative changes occur within the main classes of secondary metabolites and whether these changes are adaptively, ecologically, or selectively significant. With the rapid advancement of ecological metabolomics, this field of research may provide additional insights into the biotic and abiotic environment following a fire [26]. In light of existing knowledge gaps, this review aims to synthesize current understanding of the role of secondary metabolites in post-fire ecological memory. Specifically, we examine (i) the effects of fire on quantitative and qualitative changes in secondary metabolites, (ii) their ecological functions in post-fire environments, and (iii) their contribution to ecosystem recovery and resilience. By integrating insights from plant physiology, chemical ecology, and fire ecology, this work aims to provide a more comprehensive framework for understanding post-fire ecosystem dynamics.

This study synthesizes 43 post-fire studies to address the following questions:

How do secondary metabolites respond quantitatively and qualitatively after fire?

Do these responses reflect adaptive strategies?

Do persistent chemical changes contribute to ecosystem-level memory and successional trajectories?

## 2. Results

**Figure 1:**
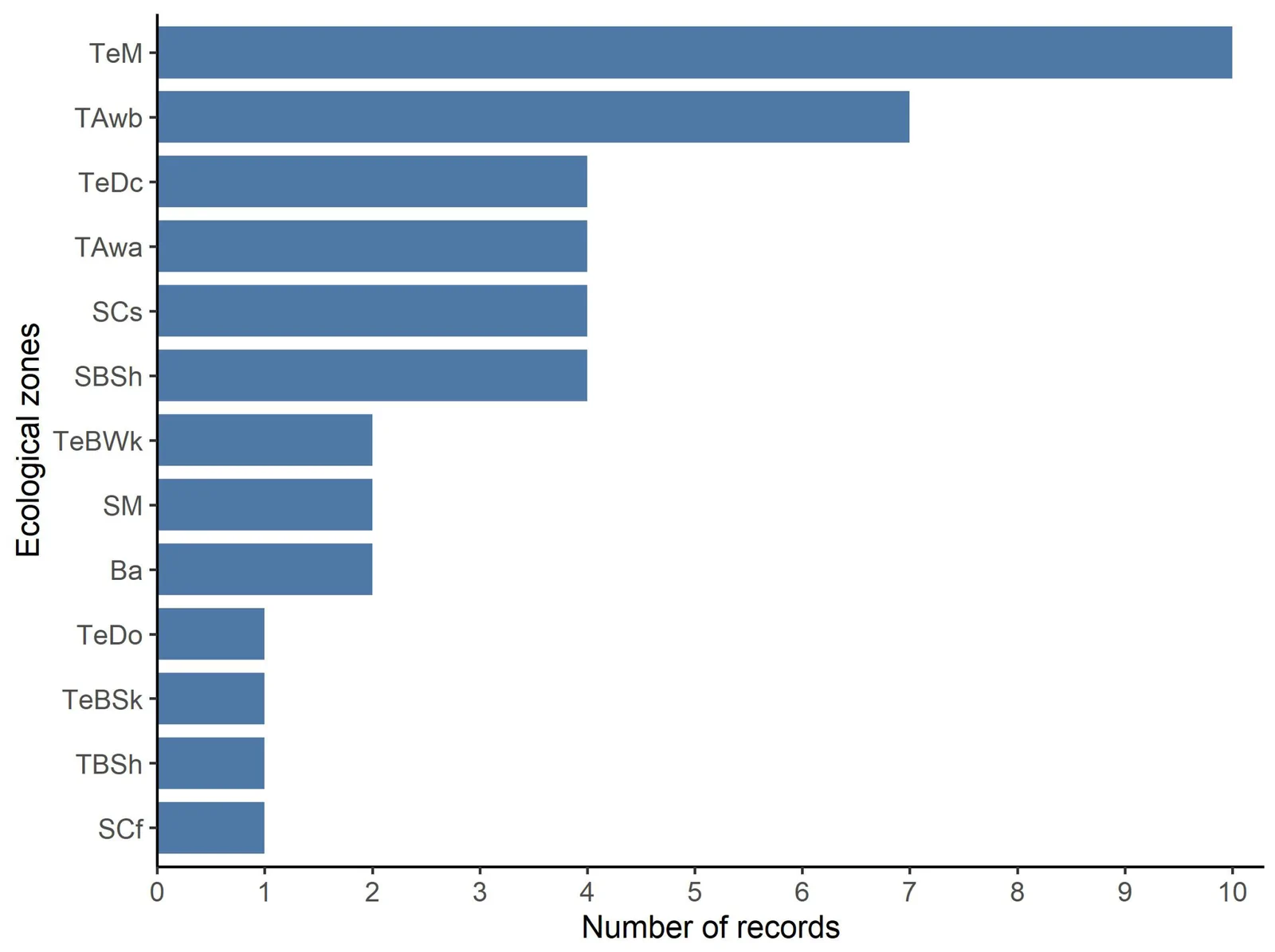
Distribution of records across ecological zones assigned to study locations according to the FAO Global Ecological Zones (GEZ 2010) classification. Abbreviations: Ba, Boreal coniferous forest; SBSh, subtropical steppe; SCf, subtropical humid forest; SCs, subtropical dry forest; SM, subtropical mountain system; TAwa, tropical moist forest; TAwb, tropical dry forest; TBSh, tropical shrubland; TeBSk, temperate steppe; TeBWk, temperate desert; TeDc, temperate continental forest; TeDo, temperate oceanic forest; TeM, temperate mountain system.

Study locations included in this review were distributed across 13 ecological zones according to the FAO Global Ecological Zones (GEZ 2010) classification [27]. Temperate mountain systems were the most represented (10 records), followed by tropical dry forests (7 records). Subtropical steppe, subtropical dry forest, tropical moist forest, and temperate continental forest were represented by four records each, whereas boreal coniferous forest, subtropical mountain systems, temperate desert, subtropical humid forest, tropical shrubland, temperate steppe, and temperate oceanic forest were represented by only one or two records each (Figure 1).

**Figure 2:**
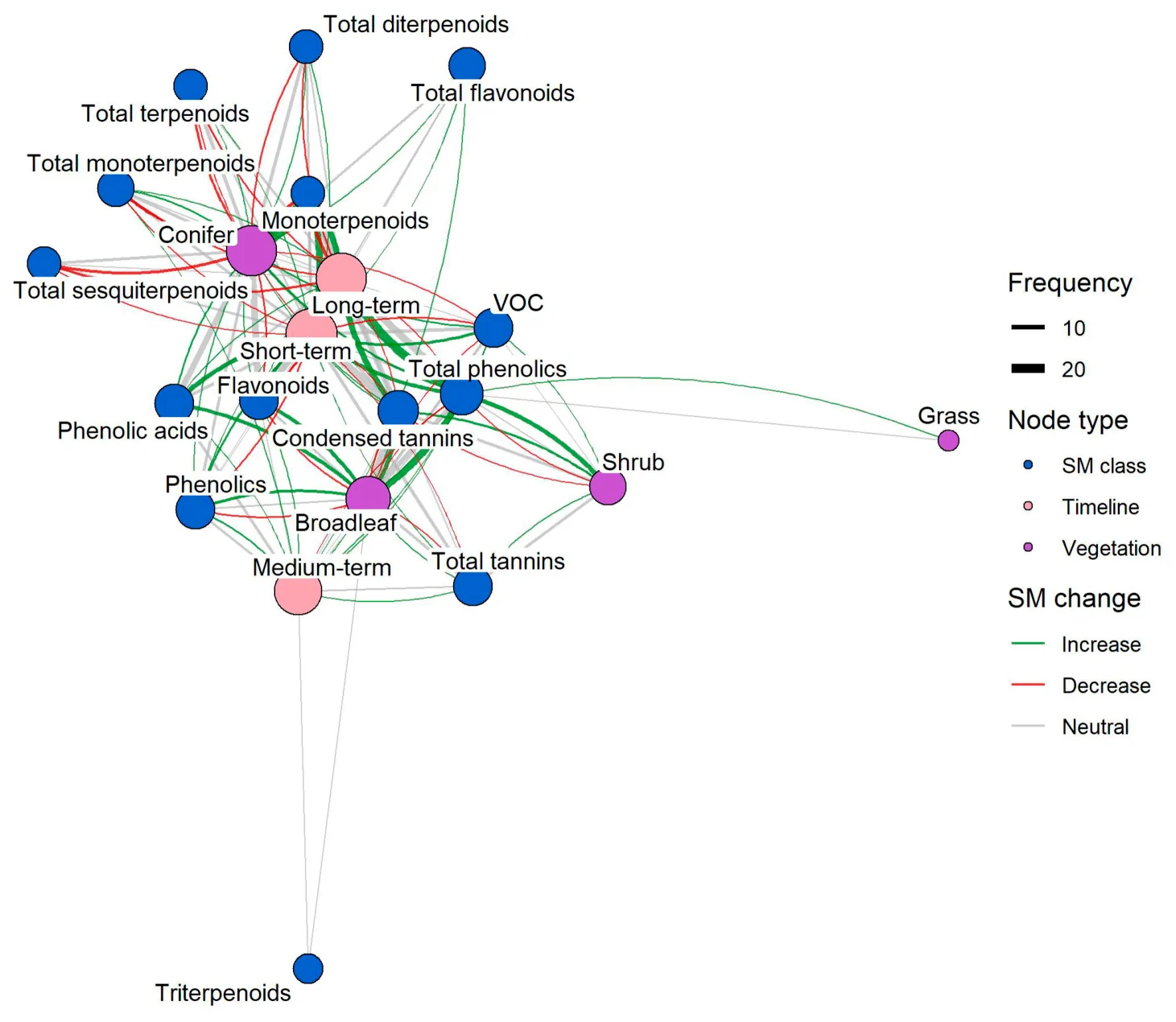
Bipartite network of post-fire secondary metabolite responses across vegetation types and post-fire timelines. Node size is proportional to degree, and node fill indicates node type (post-fire timeline, vegetation type, or secondary metabolite class). Edge color indicates the direction of metabolite response (increase, decrease, or neutral), and edge width represents the number of observations corresponding to each response direction.

The bipartite network comprised 21 nodes, including seven ecological-factor nodes (three post-fire timelines and four vegetation types) and 14 secondary metabolite classes, connected by 60 unique edges (Figure 2, Table 1). The bipartite density was 0.612, corresponding to 60 of 98 possible associations between ecological factors and metabolite classes. Together with a diameter of 4 and an average shortest path length of 1.93, this relatively high proportion of realized associations indicates a compact, interconnected network. This structure reflects the representation of many metabolite classes across multiple vegetation types and post-fire periods rather than their restriction to single ecological contexts.

Clear differences among nodes were evident in both the extent of their connectivity and the frequency with which these connections were represented in the dataset. Among ecological factors, the network showed particularly strong representation of short- and long-term post-fire responses and conifer vegetation. Short-term responses were associated with the widest range of metabolite classes (degree = 13 of 14), while both long-term responses and conifer vegetation were connected to 12 metabolite classes. These categories were also supported by the highest numbers of observations, with node strength reaching 176 for conifer vegetation, 151 for long-term responses, and 143 for short-term responses. Medium-term responses showed relatively broad connectivity (degree = 10) but substantially lower strength (35), whereas broadleaf vegetation had an intermediate degree (8) but comparatively high strength (116). Shrub vegetation (degree = 4; strength = 34) and especially grass vegetation (degree = 1; strength = 3) were more restricted in both connectivity and frequency of observations. These patterns indicate that the reviewed literature has predominantly examined phytochemical responses across short- and long-term post-fire periods and in conifer-dominated vegetation.

Among secondary metabolite classes, total phenolics showed the broadest ecological coverage, with the maximum possible degree (7) and high strength (106), indicating their representation across all vegetation-type and post-fire timeline categories. Condensed tannins similarly combined broad coverage (degree = 6) with high strength (106). Monoterpenoids showed a contrasting pattern, with the highest strength among metabolite classes (114) but a degree of only 3, indicating that numerous observations were concentrated within relatively few ecological factor categories. Flavonoids showed an intermediate pattern (degree = 5; strength = 70), as did phenolic acids (degree = 5; strength = 50) and phenolics (degree = 5; strength = 38).

The direction of post-fire responses differed among secondary metabolite classes (Table 2). Increases constituted the largest proportion of observations for total phenolics (56.6%) and monoterpenoids (49.1%), whereas neutral responses predominated for condensed tannins (62.3%), flavonoids (54.3%), total tannins (78.6%), and several less frequently represented metabolite classes. Decreases were generally less common, although they accounted for 50.0% of total sesquiterpenoid observations.

Overall, these results indicate uneven coverage of ecological contexts and phytochemical responses in the current literature on post-fire plant chemistry, with some combinations receiving broad or intensive coverage, while others remain comparatively underrepresented.

## 3. Discussion

### 3.1. Plant Secondary-Metabolite Responses to Fire

The synthesis of 43 studies shows that fire substantially alters the dynamics of secondary metabolites (SM) in forest, shrub-dominated, and grassland ecosystems, but the direction and magnitude of these changes are highly species specific and context dependent. Increases in total phenolics were frequently reported [23,25,28,29,30,31,32,33], as well as increases in specific phenolic groups, i.e., flavonoids [29], condensed tannins [23,25,30,31,34,35,36], and phenolic acids [23,24]. These changes were sometimes transient, returning to pre-fire levels within months to years [23,24], whereas longer-lasting responses were also documented [37]. Increased phenolic content has commonly been interpreted as part of the plant stress response, potentially associated with antioxidant protection [25] and defense against herbivores, particularly reduced palatability due to condensed tannins and phenolic glycosides [30,35,38]. However, altered phenolic concentrations were not consistently associated with changes in herbivory [28,39,40,41]. Neutral or decreased phenolic dynamics were also reported, demonstrating that post-fire changes depend on species, fire severity, and environmental conditions following disturbance [33,38,40,42,43,44,45,46,47,48]. Analyses of individual compounds further revealed that different phenolic constituents may respond differently even when total phenolic content remains unchanged. Total phenolics in needles of *Pinus laricio* increased shortly after fire, whereas simple phenolic acids showed a neutral response and dihydroferulic acid exhibited delayed increases [29]. Individual phenolic acids in needles of *Pinus pinaster* followed compound-specific temporal trajectories [23]. Condensed tannins, for example, may show delayed increases months or years after fire [25,35,40], although different species under the same post-fire conditions commonly show different directions of change [42,47,48]. These findings suggest that compositional changes, such as polymerization processes and redistribution among phenolic pools, may be overlooked by bulk phenolic assessment.

The analyzed literature revealed that VOCs are a common component of the fire stress response. Studies [49,50,51,52] report that acute stress signaling and protective functions are reflected in sharp increases in VOCs emissions shortly after a fire. Monoterpene concentrations in conifer species often rise significantly after fire and are associated with defense against diseases and herbivores, although these responses may decline over time [22,50,53,54,55]. Stronger emissions correlate with fire intensity, that is, higher thermal stress [50]. Some species show neutral or even reduced emissions immediately after fire, followed by recovery [49,56], while others show increases in monoterpenes, sesquiterpenes, and total essential oils [52,57,58]. These opposing responses are often linked to physiological processes such as photosynthesis and antioxidant activity [49,51,59], suggesting a close relationship between metabolism and stress. Furthermore, qualitative changes in VOCs content, such as variations in the proportion of monoterpene hydrocarbons to oxygenated molecules, indicate that fire stress causes a rearrangement of terpene biosynthesis pathways, in addition to changes in total emission [52,53]. These findings indicate that fire-induced changes in secondary metabolism cannot be interpreted as a uniform response across species or compound classes.

Fire-induced changes in secondary metabolism were also studied in relation to primary metabolism and nutrient availability. Several studies reported relationships between nitrogen availability and changes in terpenoid and phenolic production, suggesting that post-fire nutrient conditions may influence the balance between growth and chemical defense [36,58,60]. It was found that the photosynthetic process is disrupted during fire stress due to gas exchange limitations and changes in pigment composition [49,51].

Assessment of multiple species’ fire responses revealed high variability in secondary-metabolite dynamics, with neither the magnitude nor the direction of change showing consistent patterns within plant communities [25]. Plants producing higher amounts of defensive or stress-related compounds may have a competitive advantage in post-fire environments [38,61,62]. The release and transformation of these compounds reinforce the legacy of fire in ecosystem functioning by causing long-term changes in soil chemistry and nutrient cycling [63,64,65].

### 3.2. General Metabolic Context of Investigated Compounds

**Figure 3:**
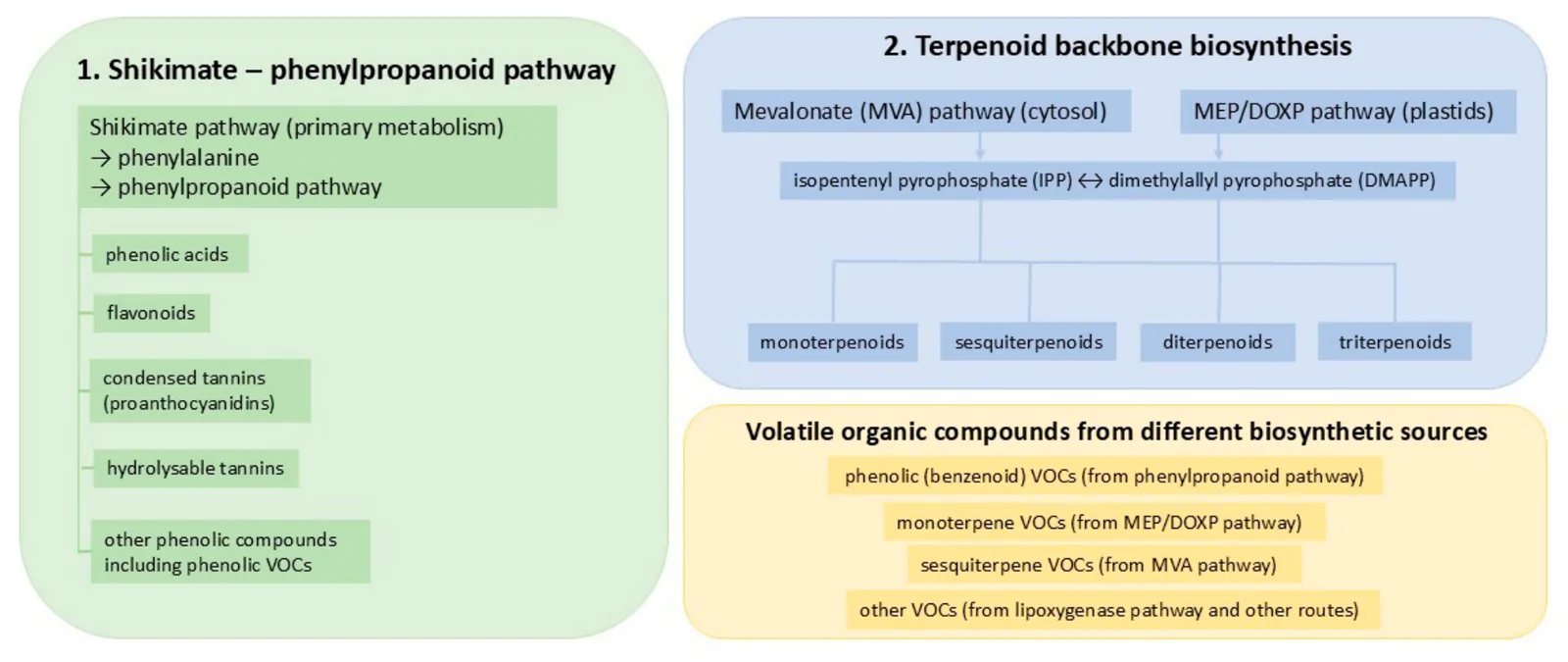
Major biosynthetic pathways underlying the production of plant secondary metabolites and VOCs.

The observed changes in secondary metabolites can be considered within the broader context of plant stress metabolism. Phenolic compounds contribute to antioxidant protection by scavenging reactive oxygen species generated under heat, dehydration, and other forms of fire-related stress [66,67], whereas flavonoids additionally provide protection against increased UV radiation following canopy loss [68]. Terpenoids, the most frequently studied group of VOCs, have both physiological (membrane stabilization under heat stress) and ecological functions, including antimicrobial and anti-herbivore defense and volatile-mediated signaling [14]. At the metabolic level, phenolic compounds are primarily associated with the shikimate/phenylpropanoid pathway [69], whereas VOCs are synthesized through multiple metabolic pathways, primarily the methylerythritol phosphate (MEP) and mevalonate (MVA) pathways for terpenoids, the lipoxygenase (LOX) pathway for green leaf volatiles, and the shikimate/phenylpropanoid pathway for benzenoid compounds [70]. The major pathways underlying the investigated metabolite groups are summarized in Figure 3. These pathways are presented as a general mechanistic context for interpreting the reviewed responses rather than as mechanisms directly demonstrated across all included studies.

### 3.3. Fire-Derived Chemical Signals

In addition to changes in endogenous plant metabolites, fire introduces chemically active compounds generated directly by combustion into the post-fire environment. Karrikins are smoke-derived butenolide compounds that act as chemical signals and can influence germination and post-fire recruitment. In the reviewed study [71], fire-derived compounds showed both stimulatory and inhibitory effects on germination, depending on compound identity and concentration, suggesting a potential role in regulating recruitment from the soil seed bank.

Karrikins are recognized as plant growth regulators, with reported effects on plant responses to abiotic stress [72,73]. The responsiveness of fire-following species to these signals may facilitate their establishment under post-fire conditions [74,75]. Because these compounds originate from combustion rather than plant biosynthesis, they were treated separately from endogenous secondary metabolites and were not included in the network analysis.

### 3.4. From Plant Responses to Ecosystem Processes

The post-fire increase in secondary metabolites, particularly phenolic compounds, is a well-established plant stress response. The abrupt stress of burning (heat, dehydration, intense light, tissue damage, and subsequent pathogen attack) induces a rapid plant response through the synthesis of metabolites, as indicated by increased gene expression and enzymatic activity in affected tissues [76]. This metabolic shift toward de novo synthesis of secondary metabolites requires carbon and energy inputs that are probably met through the mobilization and allocation of stored carbon reserves, particularly non-structural carbohydrates [77,78]. Changes in the production of secondary metabolites within individual plants influence population dynamics by improving individual resilience and increasing population diversity. Additionally, stress-induced phenotypes can be primed for a faster future response, contributing to the long-term, population-level capacity to withstand recurrent stresses [79]. These processes provide a broader, community-level framework in which post-fire dynamics of secondary metabolites influence species coexistence, trophic relationships, and community composition through various pathways, including allelopathy, signaling via volatile organic compounds, and tritrophic interactions [80,81]. Altered community composition is the universal response of fire-affected ecosystems, determined by the ability of fire-adapted species to survive fire due to anatomical, morphological, and physiological traits, as well as reproductive strategies [82]. Changes in chemical profiles also contribute to species establishment, regrowth strategies, and competitive dominance [83].

At the ecosystem level, shifts in the production of secondary metabolites can influence major ecological processes such as decomposition, nutrient cycling, soil characteristics, and overall resilience. After a fire, higher concentrations of plant-derived phenolics and terpenoids enter the litter pool, modifying its chemical composition, which can affect microbial enzymatic activity, decomposition rates, and mineralization [84,85]. Litter chemistry further influences soil processes, and plant-derived phenolic compounds remain in soil organic matter, continuing to affect decomposition and nutrient cycling over long temporal scales [84,85,86]. Fire-induced inputs of plant-derived hydrophobic organic compounds (e.g., terpenoids) can contribute to the development of soil water repellency, reducing infiltration and increasing surface runoff and erosion risk [87]. These secondary metabolites can also shape microbial community composition and function, selecting for tolerant or specialized decomposers and affecting decomposition pathways and nutrient turnover, and changes in soil processes feed back into vegetation dynamics and successional trajectories [88,89].

### 3.5. Conceptual Framework of Post-Fire Chemical Legacy

**Figure 4:**
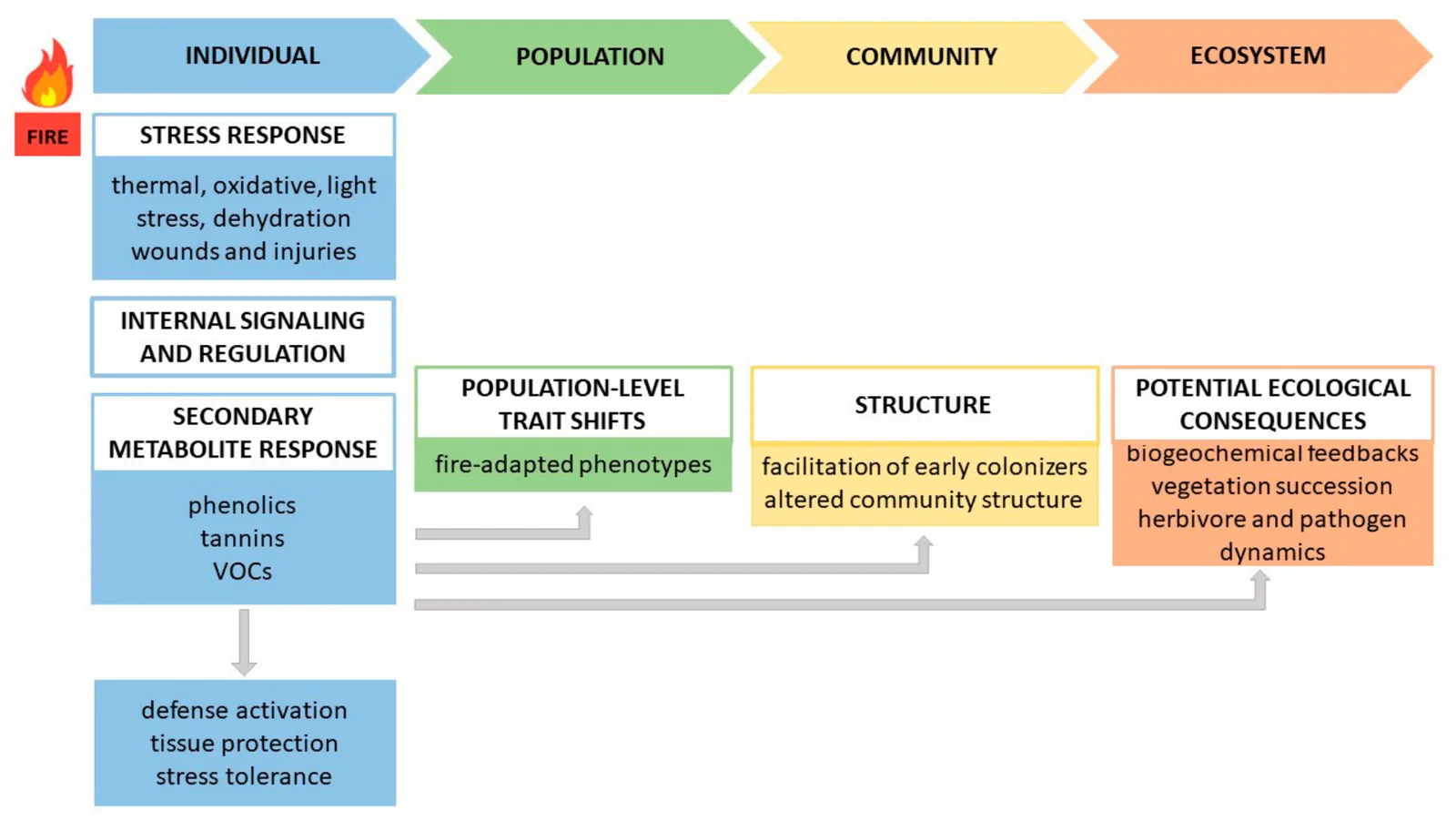
Conceptual overview of the ecological role of fire-induced secondary metabolites across hierarchical levels of biological organization.

Taken together, these results support a conceptual framework of post-fire chemical legacy (Figure 4) that integrates patterns observed in the reviewed studies with potential ecological consequences suggested by the broader literature. The short-term response is primarily characterized by rapid increases in phenolics and VOCs as part of an acute stress reaction, while metabolic reorganization of various compound classes and accumulation of persistent compounds in the environment occur over longer time scales and help stabilize altered chemical states. These chemical changes have the potential to influence species interactions, community composition, and ecosystem dynamics through their effects on herbivory, competition, regeneration, and nutrient cycling. The evidence therefore suggests a possible pathway from fire → plant physiological response → secondary metabolites → plant and community interactions → longer-term ecological legacy. However, the transition from chemical change to ecological memory requires further experimental validation.

### 3.6. Knowledge Gaps and Future Directions

Although growing evidence shows that fire triggers major changes in plant secondary metabolism, several important gaps remain. First, studies are unevenly distributed across ecological zones, limiting our ability to compare plant secondary metabolic responses to fire across contrasting ecological settings. As climate change alters fire regimes across regions, comparative studies spanning a broader range of ecological zones are needed to better assess variation in plant sensitivity to fire and post-fire recovery capacity. Second, many studies quantify total metabolite pools rather than individual compounds, limiting interpretation of functional and ecological mechanisms. Third, relatively few studies simultaneously examine fire severity, time since fire, plant traits, metabolite composition, and subsequent ecological effects. There is also a limited integration of biochemical findings into applied ecology and ecosystem management. Specifically, how variations in metabolite responses can inform fire management practices, ecosystem resilience assessments, and restoration planning remains largely unexplored. Improved insights into these areas could support the development of predictive models for post-fire recovery and guide adaptive management strategies under evolving global fire regimes. Future research should therefore combine metabolomic or targeted chemical analyses with measurements of plant performance, regeneration, species interactions, and soil processes across multiple post-fire time scales. Such approaches would help determine when observed metabolite changes represent transient physiological responses and when they contribute to persistent ecological legacies.

## 4. Materials and Methods

### 4.1. Literature Search and Study Selection

A systematic literature search was conducted in the Web of Science (WoS) and Scopus databases between 1 and 8 March 2026. The search was performed without restricting the publication period, i.e., from database inception to the date of the search. Only original research articles published in English were considered. The following search string was used in both databases:

(fire OR wildfire OR “prescribed burning”) AND (plants OR vegetation OR forest OR trees) AND (phenolics OR “phenolic compounds” OR flavonoids OR tannins OR terpenes OR terpenoids OR monoterpenes OR “secondary metabolites” OR VOCs OR “volatile organic compounds” OR karrikins OR allelochemicals).

In Web of Science, the search string was entered directly into the database search interface using the default search settings. In Scopus, the search was performed using the Title, Abstract, and Keywords fields (TITLE-ABS-KEY). No additional restrictions were applied beyond the language and document-type criteria described above.

The search yielded 1052 records from Web of Science and 1012 records from Scopus, resulting in 2064 records in total. The initial searches and recording of the retrieved references were conducted by one researcher. The results from the two databases were subsequently compared manually by copying and comparing article titles. Duplicate records were identified and removed through this manual comparison, resulting in 1055 unique records; 1009 duplicate records were therefore removed.

The resulting set of 1055 unique records was subsequently screened by two researchers based on titles, abstracts, and keywords. Screening decisions were discussed between the researchers, and any disagreements regarding eligibility were resolved by consensus. Records were excluded at this stage when they did not meet the predefined scope of the review. A total of 1012 records were excluded for the following reasons: studies not addressing the effects of wildfire or prescribed burning (n = 839); studies focused solely on fire behavior without addressing plant responses (n = 8); studies dealing with environmental pollution, including air, water, or health risks, rather than plant responses to fire (n = 106); studies not explicitly involving plants (n = 17); and studies not addressing secondary metabolites (n = 42).

Following title, abstract, and keyword screening, 43 articles remained for full-text assessment. The full texts of all 43 potentially eligible articles were obtained and assessed by the two researchers. Eligibility decisions at this stage were discussed and resolved by consensus. No additional studies were excluded during the full-text assessment; therefore, all 43 articles were included in the final qualitative synthesis. Detailed information on the studies included in the review is provided in the Supplementary Table (Table S1).

Data extraction and organization for the included studies were conducted independently by two researchers using a standardized Excel spreadsheet shared via Google Sheets. Each researcher independently extracted data from approximately half of the studies, and entries were discussed jointly during the extraction process, with any discrepancies or uncertainties resolved by consensus. The information from each study was systematically entered and organized according to predefined criteria. The following variables were recorded: (i) plant taxon studied (species, genus, or type of plant community); (ii) plant organ/tissue; (iii) SM class: phenolics, total phenolics, phenolic acids, flavonoids, total flavonoids, condensed tannins, total tannins, total terpenoids, monoterpenoids, total monoterpenoids, total sesquiterpenoids, total diterpenoids, triterpenoids, VOCs, and karrikins; (iv) direction of SM change: increased, decreased, neutral; (v) SM accumulation (long-term accumulated); (vi) fire type: prescribed fire, wildfire, or experimental burning; (vii) timespan, defined as the period between the fire event and the sampling of plant material and/or assessment of the ecological effect: short-term (immediately after the fire to 2 months), medium-term (>2 months to 1 year after the fire), long-term (>1 year after the fire), or multiple (when a study covered more than one of these periods); (viii) ecological function/ecological scale of the SM response: physiological/individual level (changes in metabolism, growth, or resource allocation); population level (plant regeneration, survival, and demography); community level (community structure and species composition); ecosystem level (biotic interactions, succession, microbial assemblages, soil processes, and nutrient cycling); (ix) statement on ecological function/level (directly measured or proposed by authors); (x) location of study. Given the heterogeneity of the included studies in research design, fire regimes, sampling strategies, plant tissues, analytical methods, and temporal scales, no standardized risk-of-bias assessment tool was applied. Instead, methodological and contextual characteristics relevant to interpreting and comparing the findings were systematically extracted and are reported in Supplementary Table S1. This approach enabled a structured synthesis of the available evidence across diverse study designs, rather than a formal grading of study quality or a quantitative assessment of risk of bias. The heterogeneity of study designs and the level of methodological detail reported in the original publications were taken into account when interpreting the findings.

Metabolites were classified according to the level of chemical characterization reported in the original studies. Categories prefixed with “total” (e.g., total phenolics, total flavonoids, total terpenoids, total monoterpenoids, total sesquiterpenoids, and total diterpenoids) included studies reporting the overall content of the corresponding metabolite class, whereas categories without the “total” prefix referred exclusively to individual metabolites identified within those classes. The condensed tannins category was an exception, as it included both measurements of total condensed tannin concentration and protein-precipitating tannins. These variables were grouped because protein-precipitating tannins largely represent the biologically active fraction of condensed tannins, although they quantify protein-binding capacity rather than total concentration.

**Figure 5:**
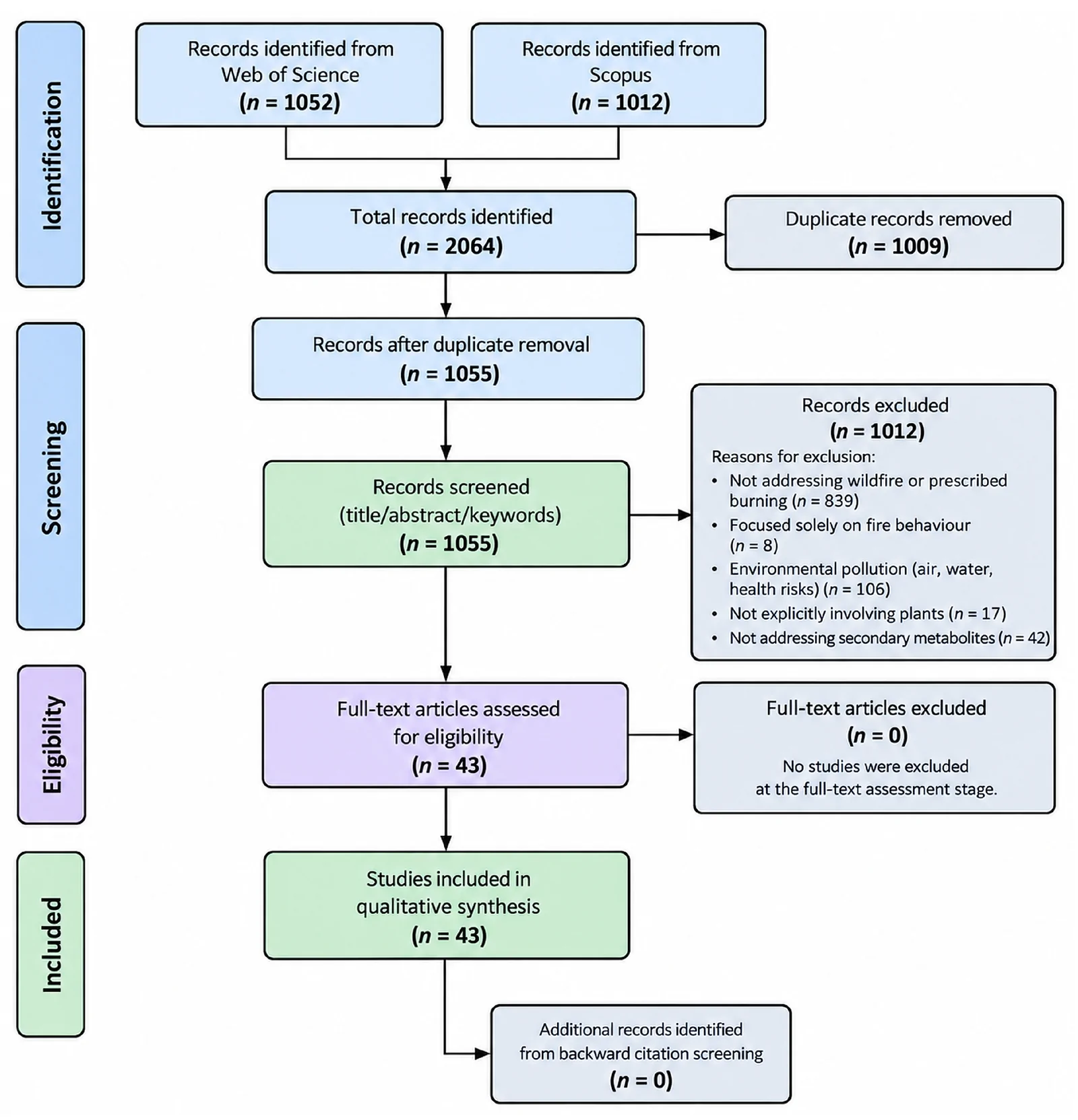
The literature search and screening process.

In addition to the database search, the reference lists of all included articles, relevant review papers, and key studies in the field were manually screened to identify potentially eligible studies that had not been retrieved through the database searches. This backward citation screening did not identify any additional eligible records. The literature search and screening process is shown in the flow diagram (Figure 5).

### 4.2. Data Processing and Analysis

All data analyses were performed using R (version 4.2.1). Study locations were classified according to the FAO Global Ecological Zones (GEZ 2010) framework [27]. Geographic coordinates reported in the original studies were spatially overlaid with the GEZ 2010 spatial dataset using the sf package (version 1.0-16) in R. When exact coordinates were not provided, ecological zones were assigned based on the reported study locality. The number of records assigned to each ecological zone was then calculated to characterize the geographic and ecological distribution of the reviewed studies.

A co-occurrence network analysis was conducted to visualize and quantify associations between ecological factors and secondary metabolite classes. Of the 43 studies included in the systematic review, only those reporting a directional secondary metabolite response relative to a pre-fire or control condition were included in the network analysis (36 studies). The basic unit of analysis was an individual record representing a plant species × sampling time × secondary metabolite class observation extracted from a study. When multiple individual compounds belonging to the same secondary metabolite class were reported for the same plant species and sampling time, each compound was treated as a separate observation. To facilitate network analysis and reduce the number of species-level categories, individual plant species were grouped into four broad vegetation categories based on their growth form and general botanical characteristics: conifers, broadleaved trees, shrubs, and grasses. Species were assigned to a single category before network analysis, and sampling times were classified into post-fire timeline categories. Each observation was also classified according to the reported direction of metabolite response (increase, decrease, or neutral). Records classified as accumulated were excluded from the network analysis because the reported information was insufficient to assign them to a specific direction of change.

The network was constructed as a bipartite network comprising two node sets: ecological factors, represented by vegetation type and post-fire timeline categories, and secondary metabolite classes. For network construction, individual observations were aggregated according to ecological factor–metabolite class combinations. Each unique vegetation type–metabolite class or post-fire timeline–metabolite class combination was represented by a single topological edge, and edge weight corresponded to the total number of observations supporting that association.

Node connectivity was characterized using degree and strength. Degree was defined as the number of distinct nodes in the opposite node set connected to a given node, reflecting the range of metabolite classes associated with each ecological factor or the range of ecological factor categories associated with each metabolite class. Node strength was calculated as the sum of the weights of all edges connected to a node and therefore represented the cumulative frequency of observations across all associations of that node. Network density was calculated as the proportion of realized associations relative to all possible associations between the two node sets. Network-level structure was further characterized by network diameter and average shortest path length, both calculated from the unweighted network topology.

Network construction and visualization were performed using the igraph (Version 2.0.3), tidygraph (Version 1.3.1), and ggraph (Version 2.2.1) packages in R.

## Figures and Tables

**Table 1:** Node-level metrics of the bipartite network linking ecological factors with secondary metabolite classes. Degree represents the number of unique connections of a node. Strength represents the cumulative frequency of observations across all edges connected to a node.

Node	Node Type	Degree	Strength
Short-term	Timeline	13	143
Conifer	Vegetation	12	176
Long-term	Timeline	12	151
Medium-term	Timeline	10	35
Broadleaf	Vegetation	8	116
Total phenolics	SM class	7	106
Condensed tannins	SM class	6	106
Flavonoids	SM class	5	70
Phenolic acids	SM class	5	50
Phenolics	SM class	5	38
VOCs	SM class	5	36
Total tannins	SM class	5	28
Shrub	Vegetation	4	34
Total monoterpenoids	SM class	4	26
Total flavonoids	SM class	4	12
Monoterpenoids	SM class	3	114
Total terpenoids	SM class	3	26
Total diterpenoids	SM class	3	22
Total sesquiterpenoids	SM class	3	20
Triterpenoids	SM class	2	4
Grass	Vegetation	1	3

**Table 2:** Distribution of post-fire secondary metabolite responses by metabolite class. Values are presented as the percentage of observations within each secondary metabolite class. Total represents the total number of observations for each metabolite class.

SM Class	Increase (%)	Decrease (%)	Neutral (%)	Total
Monoterpenoids	49.1	12.3	38.6	57
Condensed tannins	30.2	7.5	62.3	53
Total phenolics	56.6	5.7	37.7	53
Flavonoids	28.6	17.1	54.3	35
Phenolic acids	44	0	56	25
Phenolics	42.1	15.8	42.1	19
VOCs	38.9	16.7	44.4	18
Total tannins	14.3	7.1	78.6	14
Total monoterpenoids	23.1	30.8	46.2	13
Total terpenoids	15.4	30.8	53.8	13
Total diterpenoids	9.1	27.3	63.6	11
Total sesquiterpenoids	0	50	50	10
Total flavonoids	33.3	0	66.7	6
Triterpenoids	0	0	100	2

## Data Availability

No new data were created or analyzed in this study. Data sharing is not applicable to this article.

## Supplementary Materials

The following supporting information can be downloaded at: https://www.mdpi.com/article/10.3390/plants15203235/s1, Table S1: Characteristics of the 43 studies included in the literature review.
