# Supplementary material for: Exploring the Role of Secondary Metabolites in Post-Fire Ecological Memory
Source: Plants (Basel). 2026 Oct 21;15(20):3235. doi: 10.3390/plants15203235 (PMC13708834; doi:10.3390/plants15203235)
Supplement: Supplementary file 1 [file plants-15-03235-s001.zip › plants-4578522-supplementary.pdf]

Table S1. Characteristics of the 43 studies included in the literature review, including Species, Vegetation type (Veg type), Plant organ, Secondary metabolites (SM class and/or compound), Analytical methods, Direction of SM change (Dir of change), Accumulation (Accum), Ecological functions and levels (Function; Ecol level), Ecological function measured or proposed by authors (Meas/Pro), Type of fire (F type), Time since fire (F time), Study design, and Location. Abbreviations: Forest (F); Shrubland (Sh); pasture (P); Savanna (S); Woodland (W); Grassland (G); Heathland (H); Vineyard (V); prescribed burning (PB); wildfire (W); experimental burn (EB); Gas chromatograph coupled to a mass detector (GC-MS); High-performance liquid chromatography (HPLC); High-performance liquid chromatography–mass spectrometry (HPLC-MS); High-performance liquid chromatography with diode-array detection and electrospray ionization tandem mass spectrometry (HPLC–DAD–ESI–MS/MS; Increase (↑); Decrease (↓); Neutral (~); Accumulation reported (R); Accumulation not reported (NR); Directly measured (M); Proposed by authors (P).

Ref	Species	Veg type	Plant organ	SM class/compound	Analytical method	Dir of change	Accum	Function; Ecol level	Meas/Pro	F type	F time	Study design	Location
[35]	*Quercus alba* L.	F	foliage (from 20 seedlings per plot)	total condensed tannins	radial diffusion assay	~	NR	defense; ecosystem	M	PB	2-3 years	sites (3 plots within each) with different treatments: single-year burn, twice-year burn, non-burn control	Daniel Boone National Forest in eastern Kentucky, USA
[49]	*Arbutus unedo, Phillyrea latifolia, Cistus incanus, Cistus mospeliensis, Pistacia lentiscus, Quercus ilex, Quercus suber, Quercus pubescens, Myrtus communis, Pinus halepensis*	N/A	foliage	total monoterpenoids, *α*-pinene, *β*-pinene, sabinene, limonene	GC	↑↓ ~	NR	stress response; individual	M	EB	0-4 days	exposure to direct fire or to the fire-consequent wave	not specified
[23]	*Pinus pinaster* Ait.	F	foliage (1-year-old needles and control taken before burning)	total phenolics, total condensed tannins, gallic acid, protocatechuic acid, *p*-hydroxybenzoic acid, vanillic acid, syringic acid, *p*-coumaric acid, ferulic acid	protein-precipitation assay, H_2SO_4/vanillin assay, Folin-Ciocalteu, spectrofotometry, HPLC	↑ ↓ ~	NR	fire-tolerance; population	P	EB	8 days to 8 months	randomized factorial experimental design (45 trees and 5 replicates by treatment)	Cotobad (Pontevedra), northwest of Spain
[61]	*Kalmia angustifolia* L.	F	plant derived soil components	total phenolics	Swain and Hillis method		R	succession ; ecosystem	M	PB	1 to 38 years	nine Kalmia-dominated sites	east-central Newfoundland (4835¢ N, 545¢ W)
[28]	*Quercus havardii*	Sh	buds and catkins	total phenolics	Folin-Ciocalteu	~	NR	defense; ecosystem	P	PB	2 to 18 months	3 randomly selected plots: fall-burn, winter-burn and spring-burn and a fourth unburned plot	west-central Oklahoma, USA (35032'44"N, 99043'39"W; 3604'22"N, 99054'5'"W)
[52]	*Juniperus pinchotti* Sudw.	P	foliage	monoterpenes: tricyclene, *α*-pinene, camphene, *β*-pinene/sabinene, myrcene, cymene, limonene, *γ*-terpinene, terpinolene, linalool, fenchyl alcohol, camphor, citronellal, borneol, terpinen-4-ol, terpineol, carvone, bornyl acetate, total oils	GC-MS	↑↓	NR	defense; ecosystem	P	PB	11 months, 20 months	2 burn treatments: nonburned plants, 3-month-old resprouts, and 11-month regrowth	south-east of Sonora, Texas (lat 31.148N; long 100.198W)
[29]	*Pinus laricio*	F	foliage (1-year-old needles; control taken before burning)	total phenolics, vanillic acid, vanillin, syringic acid, *p*-coumaric acid, dihydroferulic acid, ferulic acid	Folin–Ciocalteu, HPLC, HPLC-MS	↑↓	NR	stress response; individual	M	PB	1 to 16 months	spring-burn plot, fall-burn plot, control plot	northwest Corsica, France (42°17'03 N, 8°55'22 E)

[57]	*Pinus nigra* subsp. *laricio* (Poir.) Maire	F	foliage (6 trees per group)	VOCs, total monoterpenoids, total sesquiterpenoids, total diterpenoids, *α*-Pinene, camphene, sabinene, b-pinene, myrcene, *α*-phellandrene, δ-3-Carene, *α*-Terpinene, *p*-Cymene, *β*-Phellandrene, limonene, (*Z*)-*β*-ocimene, (*E*)- *β*-ocimene, γ-terpinene, terpinolene, *allo*-Ocimene, estragol, thymyl methyl oxide, trans-anethol, bornyl acetate, *α*-cubebene, *α*-ylangene, *α*-copaene, *β*-bourbonene, *β*-ylangene, *β*-copaene, γ-muurolene, germacrene D, *α*-muurolene, γ-cadinene, δ-cadinene, cadina-1.4-diene, *α*-cadinene, abietic acid, dehydroabietic, neoabietic acid, palustric acid, levopimaric acid, isopimaric acid, pimaric acid, sandaracopimaric acid, pimaral, isocembrol, 4-*epi*-isocembrol, cembrene	HS-SPME, GC-FID, GC-MS, ^{13}C NMR	~ ↑	NR	defense; ecosystem	P	PB	14 months, 4 years	once-burned plots, twice-burned plots, unburned control	south-east Corsica, (418410 37N, 98120 36E)
[24]	*Pinus laricio*	F	foliage (1-, 2- and 3-year old needles)	total flavonoids, myricetin, quercetin, larycitrin, kaempferol, isorhamnetin, syringetin	$AlCl_3$ method, HPLC, HPLC-MS	↑ ~	NR	stress response; individual	M	PB	3 years	once-burned plots, twice-burned plots, unburned control	southeast Corsica, France (41 41 037 N, 9 12 36 E)
[31]	*Julbernardia globiflora* (Benth)	Wo	seedlings and resprouts (10 randomly selected per each treatment)	total phenolics total condensed tannins	Prussian blue method, butanol/HCl assay	↑	NR	defense; ecosystem	M	PB	2 and 3 years	five replicate plots at low and high fire frequency regime	Gorongosa National Park, central Mozambique (-18.998537°, 34.201478°)
[44]	*Pinus taeda* L., *Quercus stellata* Wangenh., *Q. marilandica* Muenchh.	F	foliage	total condensed tannins	protein-precipitation assay	~ ↑	NR	fire-tolerance; population	M	W	5 years	areas of high-, moderate-, low-burn severity and unburned	Bastrop State Park Bastrop County, Texas, USA (N30°6′43.992″, W97°15′38.016″)
[37]	*Cassia grandis* L., *Handroanthus heptaphyllus* (Vell.) Mattos, *Inga vera* Willd., *Ocotea diospyrifolia* (Meisn.) Mez, *Triplaris americana* L., *T. gardneriana* Wedd., *Vitex cymosa* Bertero ex Spreng., *Albizia inundata* (Mart.) Barneby and J. W. Grimes, *Genipa americana* L., *Vochysia divergens* Pohl.	F	bark (3 individuals per species)	total phenolics total tannins	Folin-Ciocalteu, hide powder (SIGMA) method		R	fire-tolerance; population	M	W	not specified, after common and frequent fire events	between-species comparison	Paraguay River, Brazil (18◦56′ 45.5"S, 57◦40′ 07.7"W)
[64]	*Pinus sylvestris* L.	F	plant derived soil components	total phenolics	Prussian blue method		R	nutrient cycling; ecosystem	M	W, PB	3, 4, 39, 67, 122, 289, 353 years	composite samples of the forest duff layer	Sweden (65°35′–66°07′N, 17°15′–19°26′E)
[65]	*Pinus ponderosa*	F	plant derived soil components	total phenolics	Prussian blue method		R	nutrient cycling; ecosystem	M	W	2 - 130 years	soil incubation experiment with plant litter extract added	Western Montana, USA (Ninemile, Clearwater, Lost Horse, Willow Creek, Sentinel Grassland)
[36]	*Colophospermum mopane*, J. Kirk ex Benth.	S	foliage	total phenolics total condensed tannins	Prussian blue method, butanol/HCl assay	↑	NR	defense; ecosystem	P	PB	1-25 years	plots with frequent fire (more than 6 fire events in 25 years, n = 27) and less frequent fire (less than 6 fire events, n = 32)	The Kruger National Park, South Africa

[71]	*Heteropogon contortus* Beauv. ex Roemer and J. A. Schultes, *Tristachya leucothrix* Trin. ex Nees	G	plant derived soil components	3-methyl-2H-furo[2,3-c] pyran-2-one<br>3,4,5-trimethylfuran-2(5H)-one	acetonitrile extraction, GC–MS	↑	NR	allelopathy; community	M	PB	20 min	samples from burnt, unburnt and adjacent soils	Ukulinga, Pietermaritzburg (29° 24′ E; 30° 24′ S)
[39]	*Eucalyptus fastigata, E. sieberi*	F	foliage	unsubstituted B-ring flavanones	HPLC-MS	↑	NR	defense; ecosystem	M	W	few days to few weeks	feeding experiments with adult leaves from the tree canopy and post-fire epicormic regrowth	Tallaganda State Forest in Southern NSW, Australia
[51]	*Cunninghamia lanceolata* (Lamb.) Hook, *Schima superba* Gardn. et Champ	N/A	air around plant	VOCs	GC-MS	↑<br>~	NR	stress response; individual	M	EB	1, 7, 30 and 90 days	exposure to gas realized from litter combustion	Fujian Province, China
[48]	*Acacia erioloba, A. haematoxylon, A. karroo, A. nilotica, A. robusta, A. sieberiana and A. xanthophloea*	N/A	stems	total condensed tannins	radial diffusion assay	↑↓	NR	defense; ecosystem	P	EB	1 month	two-factor, completely randomized experiment with simulated burning and simulated herbivory (20 plants per treatment)	arboretum at KwaZulu-Natal, South Africa (29° 37′01″ S, 30° 22′59″ E)
[38]	*Erica arborea, Erica trimera*	H	shoots (stem and leaves)	total phenolics<br>total condensed tannin	Folin–Ciocalteu, radial diffusion method	~ ↑	NR	defense; ecosystem	M	W	1 month – 3 years	plots within 3 burnt sites	Bale Mountains, Ethiopia N 06510 E 039280
[33]	*Quercus laevis* Walter, *Liquidambar styraciflua* L., *Liriodendron tulipifera* L., *Persea palustris* (Raf.) Sarg., *Acer rubrum* L.	S	belowground tissue (root crown) for 9–10 individuals per species	total phenolics	Folin–Ciocalteu		R	fire-tolerance; population	P	PB	3–4 years	6 burn compartments, which are burned on a three-year rotation	Sandhills region, North Carolina, USA (35070 N, 79100 W)
[63]	*Quercus macrocarpa*	S	foliage	total phenolics	Folin–Ciocalteu	~	NR	defense; ecosystem	M	PB	not specified, from nearly annual burns to complete fire exclusion	infestation of trees in burned and unburned areas (10 trees per treatment)	Cedar Creek Natural History Area, Minnesota, USA
[50]	*Pinus ponderosa*	F	bark and wood cores	*α*-pinene	GC-FID	↑	NR	defense; ecosystem	M	PB	14 days	ignited trees (20 per category) classified as attacked (100% scorch), unattacked (100% scorch) and unattacked (95% scorch)	Bend, Oregon, USA (44.123077 N, 121.462593 W)
[41]	*Eucalyptus rossii, E. mannifera, E. dives, E. macrorhyncha, E. radiata, E. viminalis, E. rubida E. bridgesiana*	F	foliage	formylated phloroglucinol compounds, unsubstituted B-ring flavanones	ESI-LCMS/MS, HPLC	↑ ~	NR	defense; ecosystem	M	W	4 – 6 months	post-fire epicormic leaves as feeding choices	New South Wales Southern Tablelands, Australia
[60]	*Eucalyptus dives, E. macrorhyncha, E. rossii, E. viminalis, E. rubida, E. mannifera*	F	foliage (from one tree per species per location)	formylated phloroglucinol compounds, unsubstituted B-ring flavanones	HPLC-MS, HPLC, near-infrared spectroscopy (NIRS)	↑↓ ~	NR	defense; ecosystem	P	W	9.5 months post-fire, and every 4 to 6 weeks later on up to 1 year	33 locations in the burnt landscape and 31 locations in the unburnt landscape	Macanally-Numeralla Ranges, Australia
[54]	*Pinus nigra* ssp. *Laricio., Pinus halepensis*	F	foliage, pooled sample of 5	total flavonoids, total phenolics, quercetin, larycitrin, kaempferol, syringetin, isorhamnetin,	Folin–Ciocalteu, HCL	↑↓ ~	NR	defense; ecosystem	P	PB	3 months<br>1 year<br>3 years	once-burned plots, twice-burned plots,	South-East Corsica, France (41°41’37” N;

			dominant trees (per species per plot)	total monoterpenoids, total sesquiterpenoids, total diterpenoids	treatment, spectrophotometry, GC-MS						4 years	unburned control, and adjacent burned plot featuring similar intra-zone properties	9°12’36” E); Provence, France (43°47’2” N; 5°9’13” E)
[22]	*Pinus contorta*	F	phloem below outer bark (paired sample from both south and north aspects)	monoterpenes: (+)-limonene, (−)-limonene, *α*-terpinene, (+)-*α*-pinene, (−)-*α*-pinene, myrcene, 3-carene, (+)-*β*-pinene, (−)-*β*-pinene, *α*-phellandrene, *β*-phellandrene, (+)-camphene, (−)-camphene, *γ*-terpinene, terpinolene, 4-allylanisole, (+)-pulegone, bornyl acetate, (−)-borneol, linalool, ocimene, thujone, *p*-cymene, sabinene hydrate, camphor, *α*-terpineol	GC-MS	↑↓	NR	fire-tolerance; population	P	W	2 months and 14 months	randomly chosen 40 healthy trees near the fire’s center and control was data from previous years	Jasper National Park, Alberta, Canada, (52.9844937°N, 118.0749969°W)
[62]	*Arctostaphylos uva-ursi* (L.) Spreng., *Carex geyeri* Boott	F	plant derived soil component and leaf leachates	total phenolics	Prussian Blue method	↑ ~	NR	allelopathy; community	M	W	80–140 years	microcosms and plant residual litter were collected at the end of the growing season from two plants	Sapphire mountains of the Bitterroot valley (N 4617¢37.9, W 11355¢59.7)
[45]	*Capparis erythrocarpos* var. *rosea, Acacia nigrescens, Acacia robusta, Megathyrsus maximus, Panicum infestum, P. coloratum, Urochloa mosambicensis, Digitaria eriantha*	Wo	foliage	total phenolics	not specified	↑ ~	NR	defense; ecosystem	M	PB	the study area burned every 2–3 years over 20 years; time since last fire 4 years	3 plots in 10 replicate blocks	Rift Valley Lowland Woodland, Mozambique (−18°58′, 34°20′)
[32]	*Vitis vinifera* L.	V	berries	procyanidin 1, procyanidin 2, catechin, procyanidin 3, catechin-HexA, procyanidin 4, epicatechin, *O*-methylated flavonoid-*β*-d-glucopyranoside, myricetin-3-*O*-*β*-d-glucopyranoside, quercetin-*β*-d-glucuronide, kaempferol-*β*-d-glucopyranoside, quercetin-3-*O*-*β*-d-glucopyranoside, quercetin-*β*-d-glucopyranoside, apigenin-*β*-d-glucopyranoside, kaempferol-3-*O*-*β*-d-glucopyranoside, isorhamnetin-3-*O*-*β*-d-glucopyranoside, syringetin-3-*O*-*β*-d-glucopyranoside, quercetin, isorhamnetin, syringetin, hydroxycinnamates, coutaric acid-*β*-d-glucopyranoside, caftaric acid, fertaric acid-β-d-glucopyranoside, coutaric acid, fertaric acid, *p*-coumaric acid-*β*-d-glucopyranoside, ferulic acid-*β*-d-glucopyranoside, *p*-coumaric acid, ferulic acid, phenolic acids, gallic acid, protocatechuic acid-*β*-d-glucopyranoside, protocatechuic acid, *p*-hydroxybenzoic acid-*β*-d-glucopyranoside, *p*-hydroxybenzoic, syringic acid-*β*-d-glucopyranoside, vanillic acid, benzoic acid, piceid, resveratrol, *cis*-resveratrol-*β*-d-glucopyranoside	HPLC-MS	↑	NR	N/A	N/A	EB	N/A	exposure to wildfire smoke	(located near 49.7694° N, 119.5306° W [V1] and 49.7904° N, 119.5418° W [V2])
[59]	*Protium heptaphyllum* (Aubl.) Marchand	N/A	foliage (first pair of completely expanded leaves formed after burn)	esqualene, *γ*-tocopherol, vitamin E, sitosterol, *β*-amyrin, *α*-amyrin	GC-MS	↑↓	NR	stress response; individual	P	EB	6 months	3 fire plots and control	Fazenda de Ensino, Pesquisa e Extensão, Ilha Solteira city, Brazil
[53]	*Pinus contorta* var. *latifolia* Douglas	F	phloem (50 trees per category)	(−) *α*-pinene, (+) *α*-pinene, myrcene, (−) camphene, (+) camphene, (+) 3-carene, (+) *β*-	GC-MS	↑↓	NR	defense; ecosystem	M	W	1 year	low-, moderate-, and high-burned	Yellowstone National Park, U.S.

		F		pinene, α-terpinene, (−) β-pinene, (+) limonene, β-phellandrene, terpinolene, linalool, 4-allylanisole, α-terpineol, borneol, geraniol, (−) *trans*-caryophyllene, bornyl acetate								trees and unburned control	
[56]	*Pinus ponderosa*	F	foliage	VOCs	GC-MS	↓↑	NR	stress response; individual	M	W	1-43 days	exposure to wildfire smoke	Woodland Park, Colorado, USA
[46]	*Quercus prinus* L.	F	foliage (from 10 seedlings per plot)	total condensed tannins	radial diffusion assay	↑~	NR	defense; ecosystem	M	W	6 -9 months	3 plots on burned and unburned sites	Daniel Boone National Forest in eastern Kentucky, USA
[40]	*Quercus prinus* L., *Quercus coccinea* Muenchh., *Acer rubrum*	F	foliage (two trees per species per plot)	total condensed tannins	radial diffusion assay	~↓	NR	defense; ecosystem	M	PB	1-3 months	2 plots on burned and unburned sites	Daniel Boone National Forest in eastern Kentucky, USA
[25]	*Aspidosperma quebracho-blanco* Schltdl., *Schinopsis lorentzii* (Griseb.), *Sarcomphalus mistol* (Griseb.) Hauenschild, *Atamisquea emarginata* Miers Ex Hook. & Arn), *Celtis ehrenbergiana* (Klotzsch) Liebm, *Schinus johnstonii* F.A. Barkley	F	foliage (5 individuals per species per treatment, plus control)	total phenolics, total condensed tannins	Folin–Ciocalteu, butanol/HCl assay	↑~	NR	fire-tolerance; population	M	PB	6 months to 2 years	3 experimental burns in 3 successive years; 170 individual plots within sampling site	Western Chaco Region, Argentina (28◦030 S, 64◦150 E).
[34]	*Acacia rigidula* Benth, *Prosopis glandulosa* Torr., *Celtis pallida* Torr.	G Sh	foliage	total condensed tannins	protein-precipitation assay	~↑	NR	defense; ecosystem	M	PB	6 weeks, 12 weeks, 34 weeks	randomized complete block design with 2 treatments (burned vs unburned); 5 blocks (replicates) with 2 treatments per block	north of Corpus Christi in San Patricio County, Tex. (28° 6' 49 "N, 97° 25' 00 "W)
[43]	*Rhamnidium elaeocapum* Reissek	F	bark (10-14 samples per group)	total phenolics<br>total tannins	Folin–Ciocalteu, hide powder (SIGMA) method	↑↓~	NR	fire-tolerance; population	M	W	1 to 12 years	sampling from 11 diverse forests; the grouping by last fire event and condition of aerial parts (live/dead)	Pantanal subregion Abobral, Brazil (19° 28′ 49.26'' S, 57° 00′ 57, 38'' W)
[47]	*Acacia ataxacantha, Bauhinia petersiana, Burkea africana, Combretum psidioides, Grewia flava, Philenoptera nelsii, Terminalia sericea*	S	foliage	condensed tannins,<br>total phenolics	butanol/HCl assay, Folin-Ciocalteu	↑↓	NR	defense; ecosystem	P	W	2 years and 16 years	6 replicates per species randomly in the each burned site and the control site	Waterberg Plateau Park, a Table Mountain, Namibia (2037′S, 1708′E & 2011′S, 1726′E)
[42]	*Acacia sieberiana, A. karroo, A. nigrescens, A. tortilis*	S	foliage	condensed tannins	butanol/HCl assay	~	NR	fire-tolerance; population	M	PB	4 weeks	burned and un-burned plots	Richards Bay, Ward
[58]	*Pinus sylvestris* L., *Pinus nigra* ssp. *salzmanni* (Dunal) Franco, *Pinus halepensis* Mill.	F	foliage (9-10 individuals per species per plot, control 1-year-old needles taken before burning)	total terpene, monoterpenes, sesquiterpenes, diterpenes	GC-MS	↑↓~	NR	fire-tolerance; population	P	PB	24 h and 1 year	spring-burn on 4 plots per site (3 sites)	Catalonia, Spain, Lloreda 1.5706 42.0569<br>Miravé 1.4494 41.9515<br>El Perelló 0.6816 40.9068
[30]	*Populus tremuloides*	F	foliage	phenolic glycosides (salicortin and tremulacin), condensed tannins	HPLC, butanol/HCl assay	~↑	NR	defense; ecosystem	M	W	2 years	2 plots, 4 groups per plot, categorized by burn severity:	Fishlake National Forest in central Utah, USA (38 °

												high, moderate, low, unburned	42 ′ 5 ″ N, 112 ° 49 ′ 9 ″ W).
[55]	*Melaleuca quinquenervia*	N/A	foliage	total terpenoid, *α*-pinene	GC-MS	~<br>↑	NR	defense; ecosystem	M	EB	3 months	burn treatment on 4 plants (ignited to simulate an intense fire)	N/A
